# The genome sequence of the northern bat, *Eptesicus nilssonii *(Keyserling & Blasius, 1839)

**DOI:** 10.12688/wellcomeopenres.19896.1

**Published:** 2023-08-22

**Authors:** Jeroen van der Kooij, Sonja C. Vernes, Emma C Teeling, Meike Mai, Lars Erik Johannessen, Gro Gundersen, Darwin Tree of Life Barcoding collective, Wellcome Sanger Institute Tree of Life programme, Wellcome Sanger Institute Scientific Operations: DNA Pipelines collective, Tree of Life Core Informatics collective, Darwin Tree of Life Consortium

**Affiliations:** 1 Independent researcher, Slattum, Norway; 2 School of Biology, University of St Andrews, St Andrews, Scotland, UK; 3 Neurogenetics of Vocal Communication Group, Max Planck Institute for Psycholinguistics, Nijmegen, Gelderland, The Netherlands; 4 School of Biology and Environmental Science, University College Dublin, Dublin, Leinster, Ireland; 5 Wellcome Sanger Institute, Hinxton, England, UK; 6 Natural History Museum, Oslo, Norway; 7 Akershus University Hospital, Lørenskog, Norway

**Keywords:** Eptesicus nilssonii, northern bat, genome sequence, chromosomal, Chiroptera

## Abstract

We present a genome assembly from an individual *Eptesicus nilssonii* (the northern bat; Chordata; Mammalia; Chiroptera; Vespertilionidae), derived from the placental tissue of a pregnancy that resulted a male pup. The genome sequence is 2,064.1 megabases in span. Most of the assembly is scaffolded into 26 chromosomal pseudomolecules, including the X and Y sex chromosomes. The mitochondrial genome has also been assembled and is 17.04 kilobases in length.

## Species taxonomy

Eukaryota; Metazoa; Chordata; Craniata; Vertebrata; Euteleostomi; Mammalia; Eutheria; Laurasiatheria; Chiroptera; Yangochrioptera; Vespertilionoidea, Vespertilionidae; *Eptesicus*; *Eptesicus nilssonii* (Keyserling & Blasius, 1839; Meredith *et al.*, 2011; Teeling, *et al.*, 2005) (NCBI:txid59451).

## Background

The northern bat, *Eptesicus nilssonii*, is a medium-sized, northern Palearctic bat species with a distribution ranging from Scandinavia and the Alps in the west to Kamchatka and Japan in the east. In Europe it is widely distributed in the north and east, but in central Europe it is restricted to forested areas at higher elevations ( Gerell & Rydell, 2001; López-Baucells & Burgin, 2019; Rydell, 1993; Suominen *et al.*, 2022). It has the most northern distribution of any bat species in the world, with a breeding population as far north as 69°N ( Rydell *et al.*, 1994; Speakman *et al.*, 2000). It is assessed as Least Concern for the global IUCN Red list ( Coroiu, 2016), but winter and summer census data from both Sweden and Norway indicate a recent population decline ( Ahlén & Ahlén, 2015; Eldegard *et al.*, 2021; Frafjord, 2013; Rydell *et al.*, 2019; Rydell *et al.*, 2020), and therefore the species is listed as Near Threatened in Sweden ( De Jong *et al.*, 2020) and Vulnerable in Norway ( Eldegard *et al.*, 2021). Climate change and the disuse of the insect-attracting mercury-vapour streetlights most likely play a crucial role in the decline ( Eldegard *et al.*, 2021).

Northern bats are adapted to short, light, cool summer nights and long, cold winters: they are relatively light tolerant ( Rydell, 1993). Juvenile bats undertake their first outdoor flights only two weeks after birth ( Rydell, 1992; Rydell, 1993), and animals of both sexes make extensive use of torpor in the active season ( Fjelldal *et al.*, 2023; Rydell, 1993; Siivonen & Wermundsen, 2008). During winter they express longer average bouts of torpor than other species ( Solomonov *et al.*, 2010), are found just above or sometimes even below freezing temperatures ( Masing & Lutsar, 2007; Siivonen & Wermundsen, 2008; Wermundsen & Siivonen, 2010) and have, *inter alia*, a relatively high peripheral lymphocyte count and a high vitamin E content in the liver ( Ilyukha *et al.*, 2015).

In Western Europe, nursery colonies are mainly found in buildings ( Rydell, 1993). They regularly hibernate in human-made underground sites, but recent studies have made it plausible that the majority use natural structures like screes, glacial erratics and bedrock crevices ( Blomberg *et al.*, 2021; Frafjord, 2007; Michaelsen *et al.*, 2013).

We present a chromosomally complete genome sequence for *Eptesicus nilssonii*. The sequence is based on a male placenta, retrieved shortly after birth from a monitored roost in Norway (Slattum, Nittedal municipality, Akershus county. This sampling is part of the Bat1K Project ( Teeling *et al.*, 2018) and the Darwin Tree of Life Project (DToL). The Bat1K is a collaborative effort to sequence all extant bat species, and DToL aims to sequence all named eukaryotic species in the Atlantic Archipelago of Britain and Ireland.

The use of the placenta in this context, where neither mother nor pup are euthanised, offers a more animal-friendly alternative for obtaining samples for genome analyses. Additionally, we hope that this genome may assist in uncovering the genetic basis for environmental adaptations to live in a cold climate.

## Genome sequence report

The genome was sequenced from *Eptesicus nilssonii* placental tissue collected from a monitored roost in Akershus, Norway 67 (60.02, 10.9). A total of 33-fold coverage in Pacific Biosciences single-molecule HiFi long was generated. Primary assembly contigs were scaffolded with chromosome conformation Hi-C data. Manual assembly curation corrected 49 missing joins or misjoins and removed 18 haplotypic duplications, reducing the assembly length by 0.76% and the scaffold number by 16.87%.

The final assembly has a total length of 2,064.1 Mb in 206 sequence scaffolds with a scaffold N50 of 98.0 Mb ( Table 1). Most (98.85%) of the assembly sequence was assigned to 26 chromosomal-level scaffolds, representing 24 autosomes and the X and Y sex chromosomes. Chromosome-scale scaffolds confirmed by the Hi-C data are named in order of size ( Figure 2– Figure 5; Table 2). While not fully phased, the assembly deposited is of one haplotype. Contigs corresponding to the second haplotype have also been deposited. The mitochondrial genome was also assembled and can be found as a contig within the multifasta file of the genome submission.

**Table 1.** Genome data for *Eptesicus nilssonii*, mEptNil1.1.

Project accession data		
Assembly identifier	mEptNil1.1	
Species	*Eptesicus nilssonii*	
Specimen	mEptNil1	
NCBI taxonomy ID	59451	
BioProject	PRJEB61925	
BioSample ID	SAMEA14098186	
Isolate information	mEptNil1, male: placenta (DNA sequencing, Hi-C scaffolding, RNA sequencing)	
Assembly metrics *		*Benchmark*
Consensus quality (QV)	59.2	*≥ 50*
*k*-mer completeness	100%	*≥ 95%*
BUSCO **	C:95.6%[S:93.9%,D:1.7%], F:0.8%,M:3.7%,n:12,234	*C ≥ 95%*
Percentage of assembly mapped to chromosomes	98.85%	*≥ 95%*
Sex chromosomes	X and Y sex chromosomes	*localised* *homologous* *pairs*
Organelles	Mitochondrial genome assembled	*complete single alleles*
Raw data accessions		
PacificBiosciences SEQUEL II	ERR11435996, ERR11435999, ERR11435997, ERR11435998	
Hi-C Illumina	ERR11439655	
PolyA RNA-Seq Illumina	ERR11439657, ERR11439656	
Genome assembly		
Assembly accession	GCA_951640355.1	
*Accession of alternate haplotype*	GCA_951640545.1	
Span (Mb)	2,064.1	
Number of contigs	1,629	
Contig N50 length (Mb)	2.5	
Number of scaffolds	206	
Scaffold N50 length (Mb)	98.0	
Longest scaffold (Mb)	133.11	

* Assembly metric benchmarks are adapted from column VGP-2020 of “Table 1: Proposed standards and metrics for defining genome assembly quality” from ( Rhie *et al.*, 2021). ** BUSCO scores based on the laurasiatheria_odb10 BUSCO set using v5.3.2. C = complete [S = single copy, D = duplicated], F = fragmented, M = missing, n = number of orthologues in comparison. A full set of BUSCO scores is available at https://blobtoolkit.genomehubs.org/view/mEptNil1.1/dataset/CATOCV01/busco.

**Figure 1.**
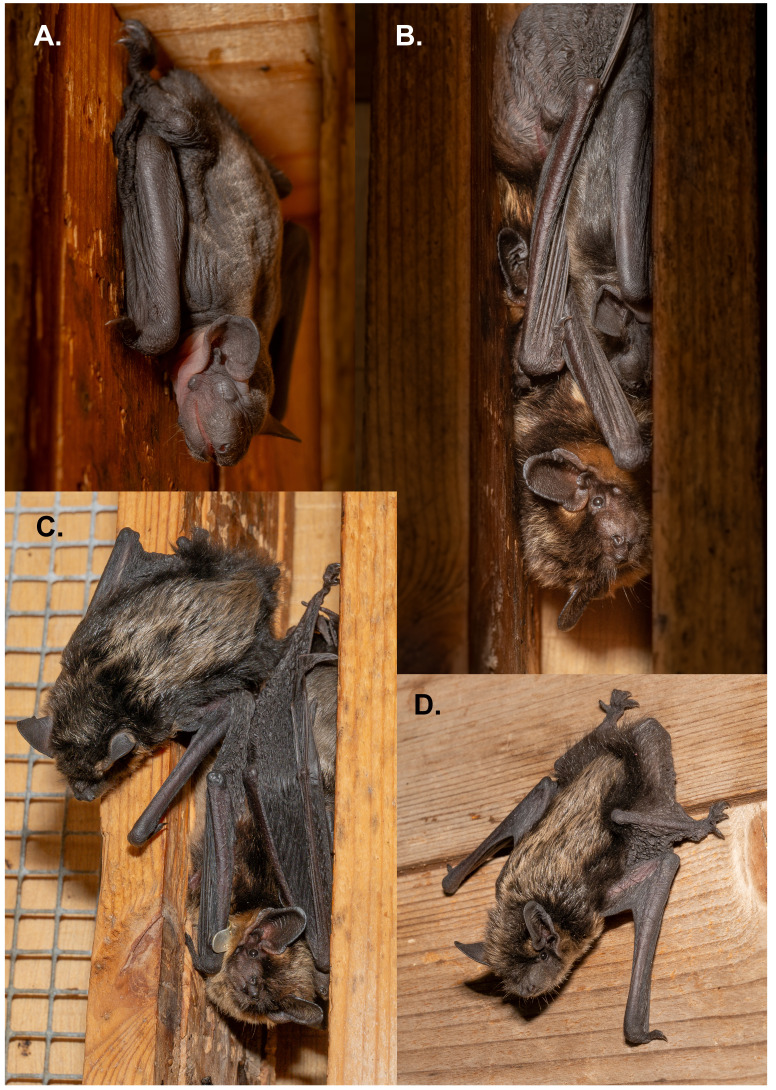
Photographs of *Eptesicus nilssonii* mother, Nikki, and her pup, Karl, captured by Jeroen van der Kooij. The placenta from Karl’s birth was sequenced for this study. **A**. Karl at 2 days, **B**. Nikki with Karl at 6 days old. **C**. Nikki with Karl at 12 days old, **D**. Karl, 12 days old.

**Figure 2.**
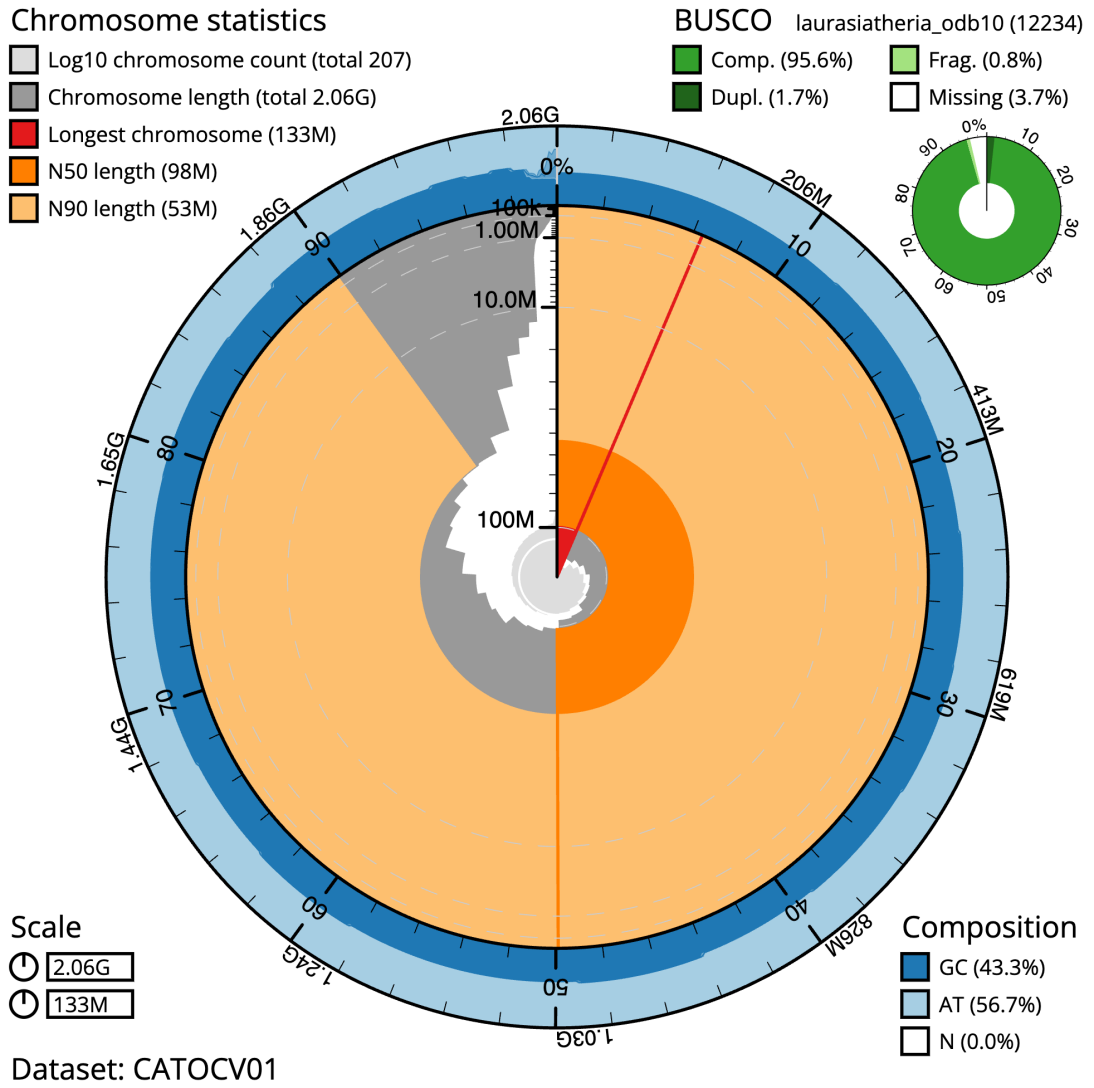
Genome assembly of *Eptesicus nilssonii*, mEptNil1.1: metrics. The BlobToolKit Snailplot shows N50 metrics and BUSCO gene completeness. The main plot is divided into 1,000 size-ordered bins around the circumference with each bin representing 0.1% of the 2,064,119,045 bp assembly. The distribution of scaffold lengths is shown in dark grey with the plot radius scaled to the longest scaffold present in the assembly (133,114,493 bp, shown in red). Orange and pale-orange arcs show the N50 and N90 scaffold lengths (98,018,206 and 52,996,688 bp), respectively. The pale grey spiral shows the cumulative scaffold count on a log scale with white scale lines showing successive orders of magnitude. The blue and pale-blue area around the outside of the plot shows the distribution of GC, AT and N percentages in the same bins as the inner plot. A summary of complete, fragmented, duplicated and missing BUSCO genes in the laurasiatheria_odb10 set is shown in the top right. An interactive version of this figure is available at https://blobtoolkit.genomehubs.org/view/mEptNil1.1/dataset/CATOCV01/snail.

**Figure 3.**
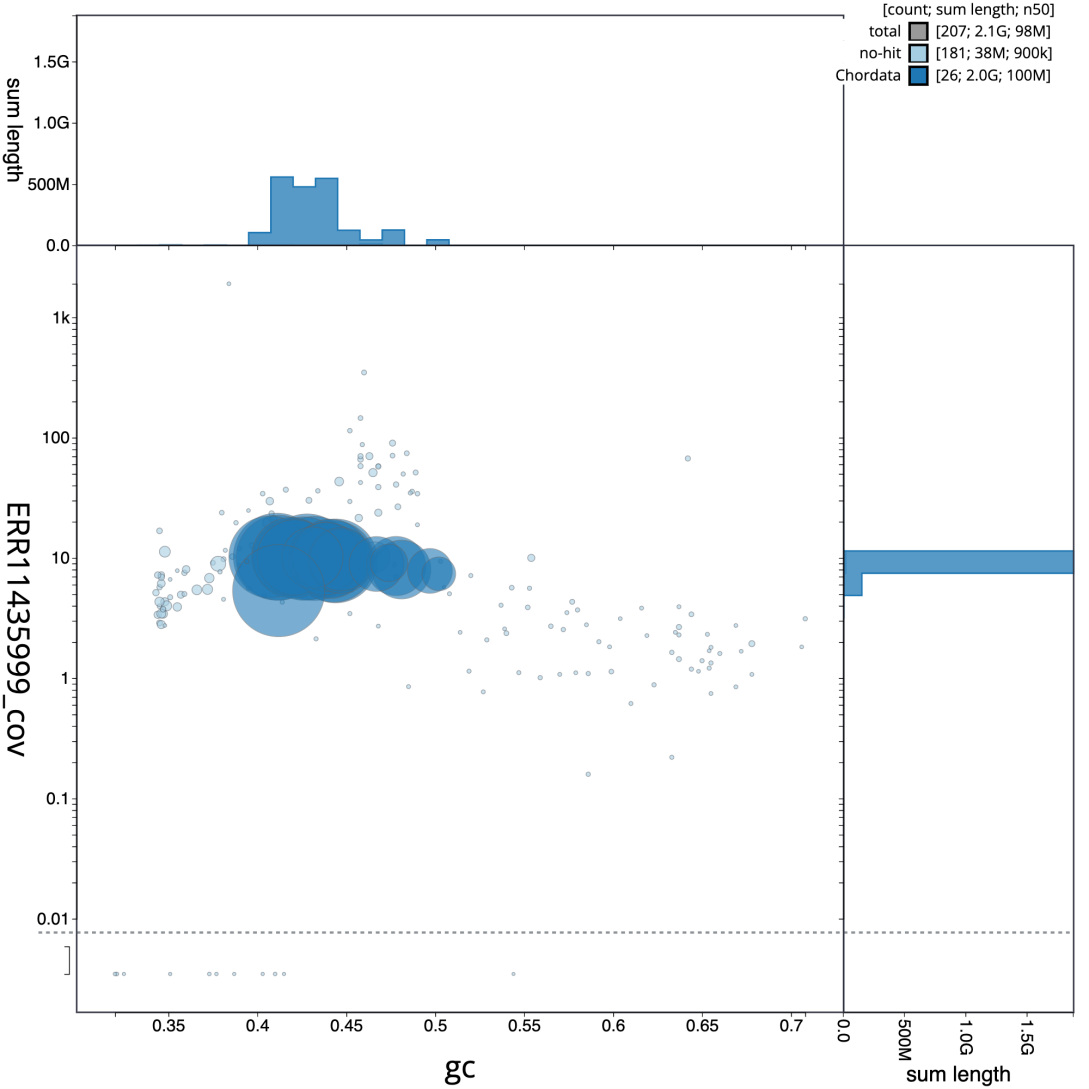
Genome assembly of *Eptesicus nilssonii*, mEptNil1.1: BlobToolKit GC-coverage plot. Scaffolds are coloured by phylum. Circles are sized in proportion to scaffold length. Histograms show the distribution of scaffold length sum along each axis. An interactive version of this figure is available at https://blobtoolkit.genomehubs.org/view/mEptNil1.1/dataset/CATOCV01/blob.

**Figure 4.**
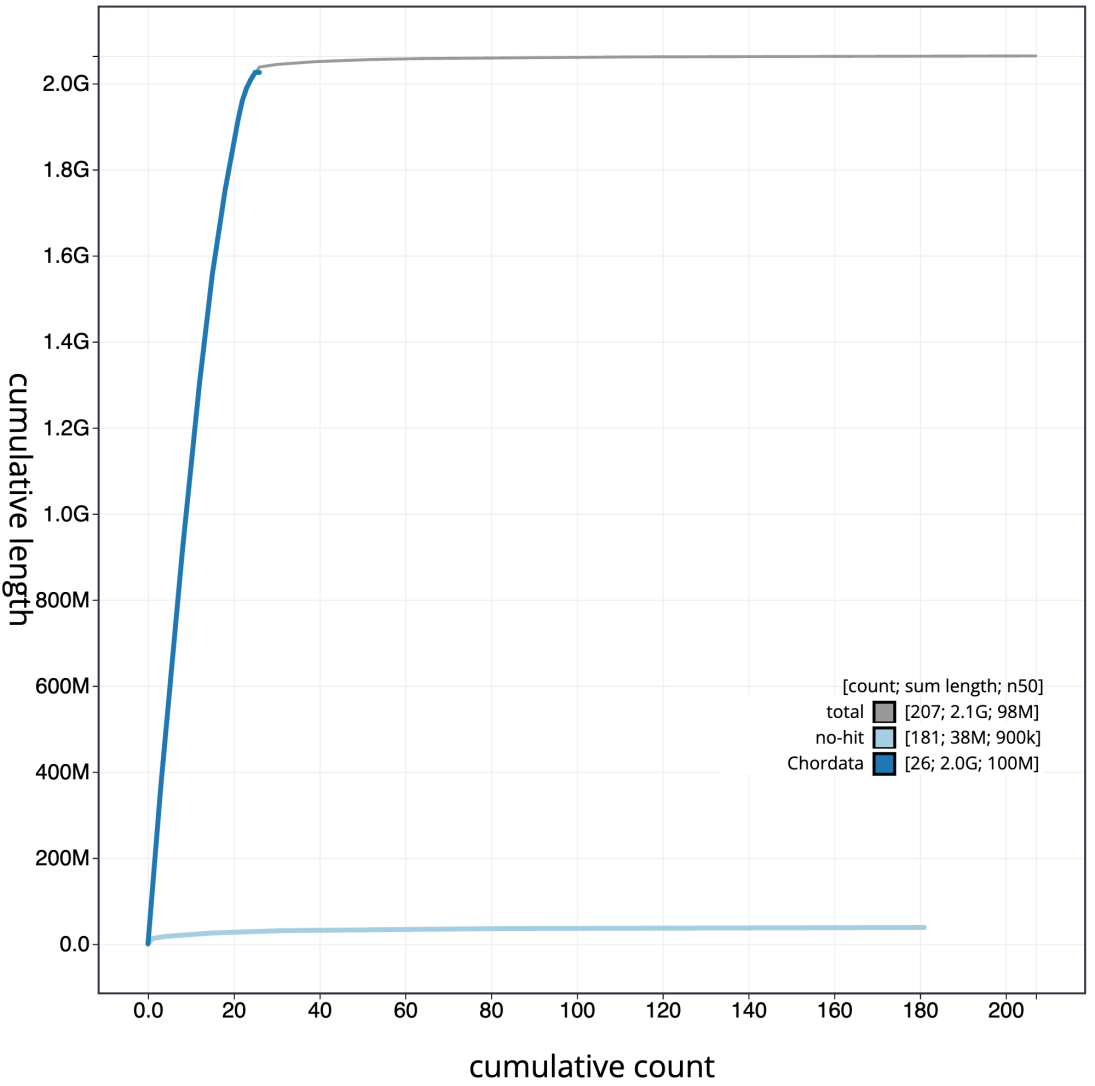
Genome assembly of *Eptesicus nilssonii*, mEptNil1.1: BlobToolKit cumulative sequence plot. The grey line shows cumulative length for all scaffolds. Coloured lines show cumulative lengths of scaffolds assigned to each phylum using the buscogenes taxrule. An interactive version of this figure is available at https://blobtoolkit.genomehubs.org/view/mEptNil1.1/dataset/CATOCV01/cumulative.

**Figure 5.**
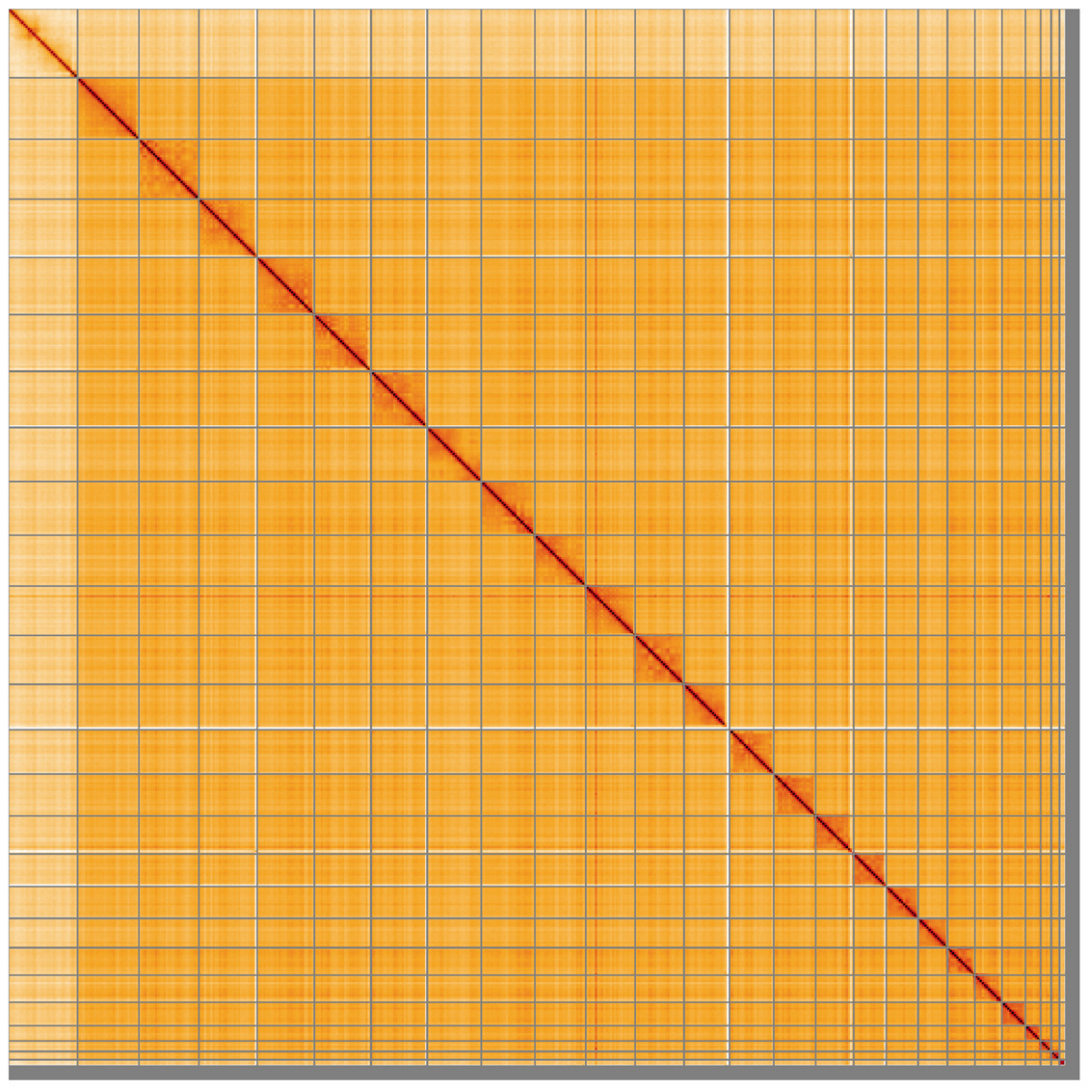
Genome assembly of *Eptesicus nilssonii*, mEptNil1.1: Hi-C contact map of the mEptNil1.1 assembly, visualised using HiGlass. Chromosomes are shown in order of size from left to right and top to bottom. An interactive version of this figure may be viewed at https://genome-note-higlass.tol.sanger.ac.uk/l/?d=X_AxuaqeQXqkZTMYsTSOwg.

**Table 2.** Chromosomal pseudomolecules in the genome assembly of *Eptesicus nilssonii*, mEptNil1.

INSDC accession	Chromosome	Length (Mb)	GC%
OX621280.1	1	118.31	41.0
OX621281.1	2	115.45	43.0
OX621282.1	3	112.64	41.0
OX621283.1	4	110.02	43.0
OX621284.1	5	109.37	44.5
OX621285.1	6	108.21	42.5
OX621287.1	8	104.28	40.5
OX621286.1	7	103.32	44.5
OX621288.1	9	98.02	42.0
OX621289.1	10	95.3	42.0
OX621290.1	11	94.12	44.0
OX621291.1	12	87.63	42.0
OX621292.1	13	85.4	44.0
OX621293.1	14	80.8	44.0
OX621294.1	15	73.27	44.0
OX621295.1	16	62.83	44.5
OX621296.1	17	61.06	44.5
OX621297.1	18	56.81	43.0
OX621298.1	19	53.0	48.0
OX621299.1	20	52.18	48.0
OX621300.1	21	45.1	46.5
OX621301.1	22	29.4	49.5
OX621302.1	23	20.08	47.5
OX621303.1	24	15.96	50.0
OX621279.1	X	133.11	41.0
OX621304.1	Y	12.64	46.5
OX621305.1	MT	0.02	38.5

The estimated Quality Value (QV) of the final assembly is 59.2 with *k*-mer completeness of 100%, and the assembly has a BUSCO v5.3.2 completeness of 95.6% (single = 93.9%, duplicated = 1.7%), using the laurasiatheria_odb10 reference set ( *n* = 12,234).

Metadata for specimens, spectral estimates, sequencing runs, contaminants and pre-curation assembly statistics can be found at https://links.tol.sanger.ac.uk/species/59451.

## Methods

### Sample acquisition and nucleic acid extraction

A placental sample from a male *Eptesicus nilssonii* pup (specimen ID SAN00002399, individual mEptNil1) was collected from Nittedal, Akershus, Norway on 2021-06-16. The specimen was taken from a maternity colony by removing the placenta from the uterus about one hour after the birth of the pup. It was placed immediately in an Eppendorf tube, and the tube was placed in dry ice in a –20°C freezer and transferred to a –80°C freezer within 24 h. The specimen was collected and identified by Jeroen van der Kooij (independent researcher).

DNA was extracted at the Tree of Life laboratory, Wellcome Sanger Institute (WSI). The mEptNil1 sample was weighed and dissected on dry ice with tissue set aside for Hi-C sequencing. The placenta tissue was cryogenically disrupted to a fine powder using a Covaris cryoPREP Automated Dry Pulveriser, receiving multiple impacts. High molecular weight (HMW) DNA was extracted using the Qiagen MagAttract HMW DNA extraction kit. HMW DNA was sheared into an average fragment size of 12–20 kb in a Megaruptor 3 system with speed setting 30. Sheared DNA was purified by solid-phase reversible immobilisation using AMPure PB beads with a 1.8X ratio of beads to sample to remove the shorter fragments and concentrate the DNA sample. The concentration of the sheared and purified DNA was assessed using a Nanodrop spectrophotometer and Qubit Fluorometer and Qubit dsDNA High Sensitivity Assay kit. Fragment size distribution was evaluated by running the sample on the FemtoPulse system.

RNA was extracted from placental tissue of mEptNil1 in the Tree of Life Laboratory at the WSI using TRIzol, according to the manufacturer’s instructions. RNA was then eluted in 50 μl RNAse-free water and its concentration assessed using a Nanodrop spectrophotometer and Qubit Fluorometer using the Qubit RNA Broad-Range (BR) Assay kit. Analysis of the integrity of the RNA was done using Agilent RNA 6000 Pico Kit and Eukaryotic Total RNA assay.

### Sequencing

Pacific Biosciences HiFi circular consensus DNA sequencing libraries were constructed according to the manufacturers’ instructions. Poly(A) RNA-Seq libraries were constructed using the NEB Ultra II RNA Library Prep kit. DNA and RNA sequencing was performed by the Scientific Operations core at the WSI on Pacific Biosciences SEQUEL II (HiFi) and Illumina NovaSeq 6000 (RNA-Seq) instruments. Hi-C data were also generated from placental tissue of mEptNil1 using the Arima2 kit and sequenced on the Illumina NovaSeq 6000 instrument.

### Genome assembly, curation and evaluation

Assembly was carried out with Hifiasm ( Cheng *et al.*, 2021) and haplotypic duplication was identified and removed with purge_dups ( Guan *et al.*, 2020). The assembly was then scaffolded with Hi-C data ( Rao *et al.*, 2014) using YaHS ( Zhou *et al.*, 2023). The assembly was checked for contamination and corrected as described previously ( Howe *et al.*, 2021). Manual curation was performed using HiGlass ( Kerpedjiev *et al.*, 2018) and Pretext ( Harry, 2022). The mitochondrial genome was assembled using MitoHiFi ( Uliano-Silva *et al.*, 2023), which runs MitoFinder ( Allio *et al.*, 2020) or MITOS ( Bernt *et al.*, 2013) and uses these annotations to select the final mitochondrial contig and to ensure the general quality of the sequence.

A Hi-C map for the final assembly was produced using bwa-mem2 ( Vasimuddin *et al.*, 2019) in the Cooler file format ( Abdennur & Mirny, 2020). To assess the assembly metrics, the *k*-mer completeness and QV consensus quality values were calculated in Merqury ( Rhie *et al.*, 2020). This work was done using Nextflow ( Di Tommaso *et al.*, 2017) DSL2 pipelines “sanger-tol/readmapping” ( Surana *et al.*, 2023a) and “sanger-tol/genomenote” ( Surana *et al.*, 2023b). The genome was analysed within the BlobToolKit environment ( Challis *et al.*, 2020) and BUSCO scores ( Manni *et al.*, 2021; Simão *et al.*, 2015) were calculated.

Table 3 contains a list of relevant software tool versions and sources.

**Table 3.** Software tools: versions and sources.

Software tool	Version	Source
BlobToolKit	4.1.7	https://github.com/blobtoolkit/ blobtoolkit
BUSCO	5.3.2	https://gitlab.com/ezlab/busco
Hifiasm	0.16.1-r375	https://github.com/chhylp123/ hifiasm
HiGlass	1.11.6	https://github.com/higlass/higlass
Merqury	MerquryFK	https://github.com/ thegenemyers/MERQURY.FK
MitoHiFi	3	https://github.com/marcelauliano/ MitoHiFi
PretextView	0.2	https://github.com/wtsi-hpag/ PretextView
purge_dups	1.2.5	https://github.com/dfguan/purge_ dups
sanger-tol/genomenote	v1.0	https://github.com/sanger-tol/ genomenote
sanger-tol/readmapping	1.1.0	https://github.com/sanger-tol/ readmapping/tree/1.1.0
YaHS	1.2a.2	https://github.com/c-zhou/yahs

### Wellcome Sanger Institute – Legal and Governance

The materials that have contributed to this genome note have been supplied by a Tree of Life collaborator. The Wellcome Sanger Institute employs a process whereby due diligence is carried out proportionate to the nature of the materials themselves, and the circumstances under which they have been/are to be collected and provided for use. The purpose of this is to address and mitigate any potential legal and/or ethical implications of receipt and use of the materials as part of the research project, and to ensure that in doing so we align with best practice wherever possible. The overarching areas of consideration are:

•   Ethical review of provenance and sourcing of the material

•   Legality of collection, transfer and use (national and international)

Each transfer of samples is undertaken according to a Research Collaboration Agreement or Material Transfer Agreement entered into by the Tree of Life collaborator, Genome Research Limited (operating as the Wellcome Sanger Institute) and in some circumstances other Tree of Life collaborators.

## Data Availability

European Nucleotide Archive: *Eptesicus nilssonii* (northern bat). Accession number PRJEB61925; https://identifiers.org/ena.embl/PRJEB61925. ( Wellcome Sanger Institute, 2023) The genome sequence is released openly for reuse. The *Eptesicus nilssonii* genome sequencing initiative is part of the Darwin Tree of Life (DToL) project. All raw sequence data and the assembly have been deposited in INSDC databases. The genome will be annotated using available RNA-Seq data and presented through the Ensembl pipeline at the European Bioinformatics Institute. Raw data and assembly accession identifiers are reported in Table 1.

## Author information

Members of the Darwin Tree of Life Barcoding collective are listed here: https://doi.org/10.5281/zenodo.4893703.

Members of the Wellcome Sanger Institute Tree of Life programme are listed here: https://doi.org/10.5281/zenodo.4783585.

Members of Wellcome Sanger Institute Scientific Operations: DNA Pipelines collective are listed here: https://doi.org/10.5281/zenodo.4790455.

Members of the Tree of Life Core Informatics collective are listed here: https://doi.org/10.5281/zenodo.5013541.

Members of the Darwin Tree of Life Consortium are listed here: https://doi.org/10.5281/zenodo.4783558.
