# Supplementary material for: The genome sequence of the northern bat, Eptesicus nilssonii (Keyserling & Blasius, 1839)
Source: Wellcome Open Res. 2023 Aug 22;8:362. [Version 1] doi: 10.12688/wellcomeopenres.19896.1 (PMC11106602; doi:10.12688/wellcomeopenres.19896.1)
Supplement: Peer review history

# Reviewer report: Reviewer response for version 1

**DOI:** 10.21956/wellcomeopenres.22031.r83703

**Published:** 2024-06-11

**Authors:** Wenhua Yu, Xiaoyun Wang

**Affiliations:** 1 Guangzhou University, Guangzhou, China; 2 School of Life Sciences, Guangzhou University (Ringgold ID: 47875), Guangzhou, Guangdong, China

The authors have successfully obtained the genome of the *Eptesicus nilssonii *using the placental tissue. Given the current relative scarcity of complete genomic data within the Chiroptera, this study undoubtedly provides meaningful genomic information for subsequent research. 

 The manuscript is well-written, and the analytical methods used are all reasonable and high standard, serving as an ideal framework for future genomic report-like research outcomes. At the current stage, I only recommend that the authors further describe the introduction regarding the species' adaptive evolution to low temperatures at the genomic level, which would also lay the foundation for future research and reporting.

 minor issue:

 In Table 2, an error in the sequencing 7 and 8.

Are sufficient details of methods and materials provided to allow replication by others?

Yes

Is the rationale for creating the dataset(s) clearly described?

Yes

Are the datasets clearly presented in a useable and accessible format?

Yes

Are the protocols appropriate and is the work technically sound?

Yes

Reviewer Expertise:

bats, phylogeny, taxnomony, speciation

We confirm that we have read this submission and believe that we have an appropriate level of expertise to confirm that it is of an acceptable scientific standard.

# Reviewer report: Reviewer response for version 1

**DOI:** 10.21956/wellcomeopenres.22031.r83706

**Published:** 2024-06-11

**Authors:** Keping Sun

**Affiliations:** 1 Northeast Normal University, Changchun, Jilin, China

This manuscript reports the first genome sequence of the northern bat, *Eptersicus nilssonii*, which is useful for studying the environmental adaptations of this species. In particular, this study used placental tissue for genome sequencing, providing an animal-friendly alternative for obtaining samples for genome analyses. The tables and figures are clear, and the manuscript is easy to read. However, I still have some minor comments for the manuscript.

 (1) In the manuscript, when *Eptesicus nilssonii* first appears, write the full name, and for subsequent mentions, provide the abbreviation, E. nilssonii.

 (2) For the first sentence “The genome was sequenced from *Eptesicus nilssonii* placental tissue collected from a monitored roost in Akershus, Norway 67 (60.02, 10.9).” in “Genome sequence report”, I think you can remove it to the fourth paragraph and integrate with the second sentence. Please indicate N and E.

 (3) For Figures 3 and 4, I think you can rotate the description of the y-axis counterclockwise by 180 degrees.

 (4) For the reference “Siivonen Y, Wermundsen T…”, page is absent.

Are sufficient details of methods and materials provided to allow replication by others?

Yes

Is the rationale for creating the dataset(s) clearly described?

Yes

Are the datasets clearly presented in a useable and accessible format?

Yes

Are the protocols appropriate and is the work technically sound?

Yes

Reviewer Expertise:

Comparative genomics, molecular adaptation, speciation

I confirm that I have read this submission and believe that I have an appropriate level of expertise to confirm that it is of an acceptable scientific standard.

# Reviewer report: Reviewer response for version 1

**DOI:** 10.21956/wellcomeopenres.22031.r83705

**Published:** 2024-05-20

**Authors:** Xiuguang Mao

**Affiliations:** 1 East China Normal University, Shanghai, China

van der Kooij et al. present a high-quality chromosome-scale assembly for a male *Eptesicus nilssonii*. The processes of genome sequencing, assembly and assessment follow a high standard pipeline. The total number of chromosomal-level scaffolds corresponds to the haploid chromosome number of this species (2n=50). This reference genome, together with another one from a female individual (GenBank accession: GCA_030846915.1), will provide valuable resources to study the genetic basis of cold adaptation.

 Three minor comments:

1. Can authors say a little bit about how they assign the sex chromosomes (X and Y chromosome)?
2. Can authors provide specific amounts of sequencing data for HiFi data (? Gb) and Hi-C data (? Gb) used in genome assembly?
3. In Column “Chromosome” in Table 2, “8” and “7” should be reordered if chromosomes are named in the order of size.

Are sufficient details of methods and materials provided to allow replication by others?

Yes

Is the rationale for creating the dataset(s) clearly described?

Yes

Are the datasets clearly presented in a useable and accessible format?

Yes

Are the protocols appropriate and is the work technically sound?

Yes

Reviewer Expertise:

Comparative genomics, genome assembly, speciation, chromosome evolution, bats

I confirm that I have read this submission and believe that I have an appropriate level of expertise to confirm that it is of an acceptable scientific standard.

# Reviewer report: Reviewer response for version 1

**DOI:** 10.21956/wellcomeopenres.22031.r66152

**Published:** 2023-09-04

**Authors:** Graham Etherington

**Affiliations:** 1 Earlham Institute, Norwich, UK

The authors describe the first genome sequence of the northern bat, *Eptesicus nilssonii *. The paper is well-written and although quite concise, easy to follow. As one of the rationales for this work is to assist with uncovering the genetic basis of environmental adaptations to cold climates, I suggest they cover this in their introduction (see point 3 below). Other than this, I only have a couple of corrections and suggestions.

 Background

1. Close bracket after "Akershus county".
2. The authors state that the sampling of *Eptesicus nilssonii *is part of DToL, so they should specify the status of this species in the UK (i.e. vagrant).
3. The authors state that they hope the genome may assist in uncovering the genetic basis for environmental adaptations to live in a cold climate. As this is one of their key aims, they should cover some previous work around this in the preceding paragraphs.

Genome sequence report

1. The second sentence doesn't read very well and should be changed (e.g. "A total of 33-fold coverage of Pacific Biosciences single-molecule HiFi long-read data was generated").

Are sufficient details of methods and materials provided to allow replication by others?

Yes

Is the rationale for creating the dataset(s) clearly described?

Partly

Are the datasets clearly presented in a useable and accessible format?

Yes

Are the protocols appropriate and is the work technically sound?

Yes

Reviewer Expertise:

Bioinformatics, genome assembly, conservation genomics.

I confirm that I have read this submission and believe that I have an appropriate level of expertise to confirm that it is of an acceptable scientific standard.
